# The Book World of Medicine and Science

**Published:** 1895-03-09

**Authors:** 


### THE BOOK WORLD OF MEDICINE AND SCIENCE.

ON PRESERVATION OF HEALTH IN INDIA. By Sir J. FAYRER, K.C.S.I., M.D., F.R.S., President of the Medical Board at the India Office. (London: Macmillan and Co. 1894. pp. 51. Price 1s.)

This little brochure contains the substance of a lecture delivered to the students of Cooper's Hill College. It commences with a brief account of the geographical boundaries of British India, followed by a few remarks on the varieties of climate found in the vast extent of that country. The climatic difficulties to contend against are, "chiefly, those due to extremes of temperature, dryness, moisture, and miasmata." "If," writes the author, "the question be asked how a young man should live with the view to preserve his health in India, the answer is that he should live temperately in all things, always wear woollen, however light, next his person, avoid exposure to the direct rays of the sun and notoriously miasmatic localities, go to bed and rise early, eat moderately and at regular hours, smoke and drink as little as possible, and guard against giving way to passion, excitement, or the irritability of temper so easily acquired in hot climates. He should pay attention immediately to all tendency to bowel complaint or other acute symptoms, avoid idleness and its consequent *ennui* on the one hand, and overwork, mental or physical, on the other." All this is doubtless most excellent advice, but it may be feared that many young men could not follow it, and would not if they could. Avoidance of exposure is not always in the power of every person, for work must be done whether the vertical sun shines fiercely, or the frost of Upper India appears colder than that of Europe. And the carelessness of young people, both male and female, in India exceeds even that of young people at home. They will not "study well the clime," nor endeavour to "mould its manners to their obsequious forms." In the pride of a fleeting youth and passing strength, although they have probably read the classics, in which there are half a dozen instances showing there are desires the gratification of which is fatal, they do not profit thereby. Neither do they profit by the advice given them in various books on the subject. Let us hope that Sir J. Fayrer's little work will be more favourably received. The author also alludes to the importance of commencing Indian life in the cold season. He gives advice as regards hats, clothing, diet, drinking water, houses, and plain directions as to what should be done if a person unfortunately contracts one of those maladies so prevalent in the tropics, viz., malaria, dysentery, cholera, or sun stroke. Of course, being an authority on the subject, he gives advice respecting snake-bite. In short, the lecture, as it now appears, is *multum in parvo*, which every one proceeding to India should obtain and study.

